# Emergent method for management of splenic artery aneurysms rupture: A case report

**DOI:** 10.1016/j.ijscr.2024.109406

**Published:** 2024-02-24

**Authors:** Javad Salimi, Parham Nikraftar, Fatemeh Rashidi, Mohammadreza Azimi, Amir Shokri

**Affiliations:** a Department of Vascular Surgery, Sina Hospital, Tehran University of Medical Science, Tehran, Iran; b School of Medicine, Tehran University of Medical Sciences, Tehran, Iran; c School of Medicine, Mazandaran University of Medical Sciences, Sari, Iran; d Department of General Surgery, School of Medicine, Tehran University of Medical Sciences, Tehran, Iran

**Keywords:** Splenic artery aneurysm, Hybrid, Endovascular, Open surgery, Rupture of aneurysm

## Abstract

**Introduction and importance:**

Although endovascular therapy is becoming more used for the treatment of splenic artery aneurysms (SAAs) instead of open surgery, there is limited information available on the emergent hybrid approach, selectively. We present our experience of hybrid therapy using an emergent endovascular balloon for inflow control and open resection.

**Case presentation:**

A 34-year-old woman was brought to the emergency room after it was reported that she had a pseudoaneurysm in her splenic artery at a different medical facility. The patient was hemodynamically stable. Then we underwent a combination of endovascular and open procedures, using balloon proximal control and open aneurysm resection. She was discharged from hospital on the fifth postoperative day after the operation.

**Clinical discussion:**

There is no agreement on how to treat SAA patients. Endovascular procedures such as endovascular intervention are also being used, minimizing the risks of surgery and shortening the patient's hospital stay, but complications remain. We propose to try SAA's emergency hybrid strategy operation with a good prognosis and fewer complications.

**Conclusion:**

It seems that, compared to open surgery alone when endovascular procedures were impossible, elective hybrid procedures are more secure and efficient in stable patients and could make the operation easier without more dissection for proximal control of splenic artery.

## Highlights

• Efficacy of a hybrid approach involving both endovascular and open procedures for the treatment of splenic artery aneurysms (SAAs)
• Emergency Hybrid Procedure
• This approach is considered a viable alternative to more invasive procedures, highlighting the importance of maintaining collateral circulation in the spleen.

## 1 Introduction

Splenic artery aneurysms (SAAs) are rare conditions that can be life-threatening, but prompt detection and intervention are essential [1,2].

It has been determined that the incidence rates of visceral artery aneurysms, an uncommon vascular pathology, vary between 0.01 % and 0.2 % [3,4].

After abdominal aortic aneurysms and iliac artery aneurysms, splenic artery aneurysms are the third most common type of intra-abdominal aneurysm [5]. According to reports, between 2 % and 10 % of patients experience rupture of SAAs as their first presentation [6].

The most prevalent approach for treating SAAs are with medication, close observation, laparoscopic surgery, open abdominal surgery, and endovascular therapy (stent or coil embolization) [7,8].

Depending on the comorbidities and general state of health of the patient, surgery used to be the most common method. Currently, angiography has become the primary method for treating most visceral aneurysms because of the advancements in interventional angiography [9].

Although other studies indicated endovascular treatments were thought to be the initial line of treatment for splenic artery aneurysms, the majority of the time, open surgery is used to address problems or to repair a lack of endovascular treatments, an allergy to contrast medium, or a failure of endovascular techniques. Splenectomy, aneurysmectomy, or splenic artery ligation are possible surgical treatments for splenic artery aneurysms [8].

Elective hybrid therapy has been used to treat splenic artery aneurysm [10], but emergent hybrid therapy, which uses an emergent endovascular balloon for inflow control and open resection, is not yet reported.

The current study provides specifics on the effective emergency treatment of a patient suffering from splenic artery aneurysms.

## 2 Case presentation

A 34-year-old single Iranian woman arrived at the emergency department complaining of the sudden onset of sharp abdominal pain from the previous day with no history of recent trauma.

The epigastric region was identified as the origin of the abdominal pain that radiated backward. Importantly, this pain was consistent in its location, unaffected by body position, and exhibited no variation in intensity following meals.

**Fig. 1:**
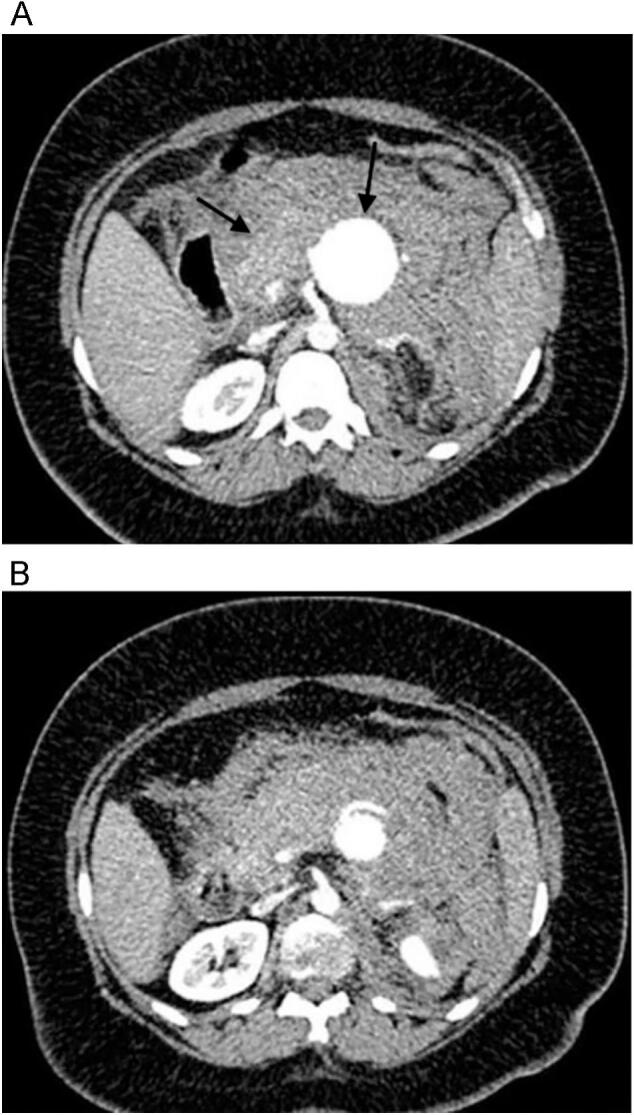
Pretreatment contrast-enhanced CT is showing an aneurysmatic dilation in the proximal of the splenic artery. Arrows: Aneurysm sac and retroperitoneal hematoma.

It is important to highlight that the patient initially sought medical attention at another healthcare facility. The ultrasound examination disclosed a proximal aortic aneurysm. Afterwards, a computed tomography angiography (CTA) revealed a splenic artery pseudoaneurysm and a left gastric artery pseudoaneurysm or aneurysm at increased risk. Due to the limited available facilities, the patient was subsequently admitted to our hospital (Fig. 1).

She suffered from nausea without vomiting. Hematochezia, melena, anorexia, or fever were not present. Her past medical history was unremarkable. He was a non-smoker and non-alcoholic. Physical examination revealed 120/75 mmHg blood pressure, a temperature of 37 °C, and heart and respiratory rates of 110 beats and 13 breaths per minute, respectively.

Examining the patient's abdomen is not possible due to the possibility of aneurysms.

The complete blood count (CBC) reported white cell and platelet counts of 20.23 × 10^3^/L and 404 × 10^3^/L, respectively. Hemoglobin was 11 g/L at admission. C-reactive protein (CRP) was 51 mg/dL. The patient was stable. The work has been reported in line with the SCARE criteria [11]. We conducted an emergent hybrid procedure.

The patient was urgently taken to the catheterization laboratory for endovascular intervention. In light of the challenging anatomical position of the aneurysm, traditional methods like inserting a stent graft or coiling the proximal normal artery were deemed impractical. Coiling the aneurysm sac alone was also ruled out due to the rupture. Given the success of this approach in elective cases previously, we decided to manage with a similar treatment strategy.

**Fig. 2:**
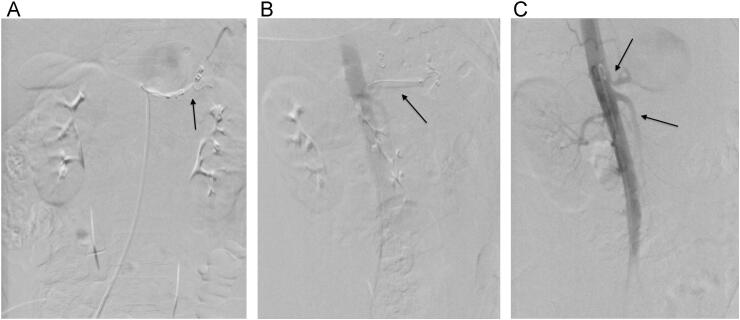
a: Coil and Distal embolization, b: the balloon control, c: celiac and SMA, the first angiography with pigtail catheter.

During the procedure, a deflated balloon was inflated to a fixed size of 7 * 40 mm as proximal control after being attached to a catheter that was inserted into the splenic artery right before the aneurysm over a guidewire. A distal interlock coil was used to attain distal occlusion (Fig. 2).

**Fig. 3:**
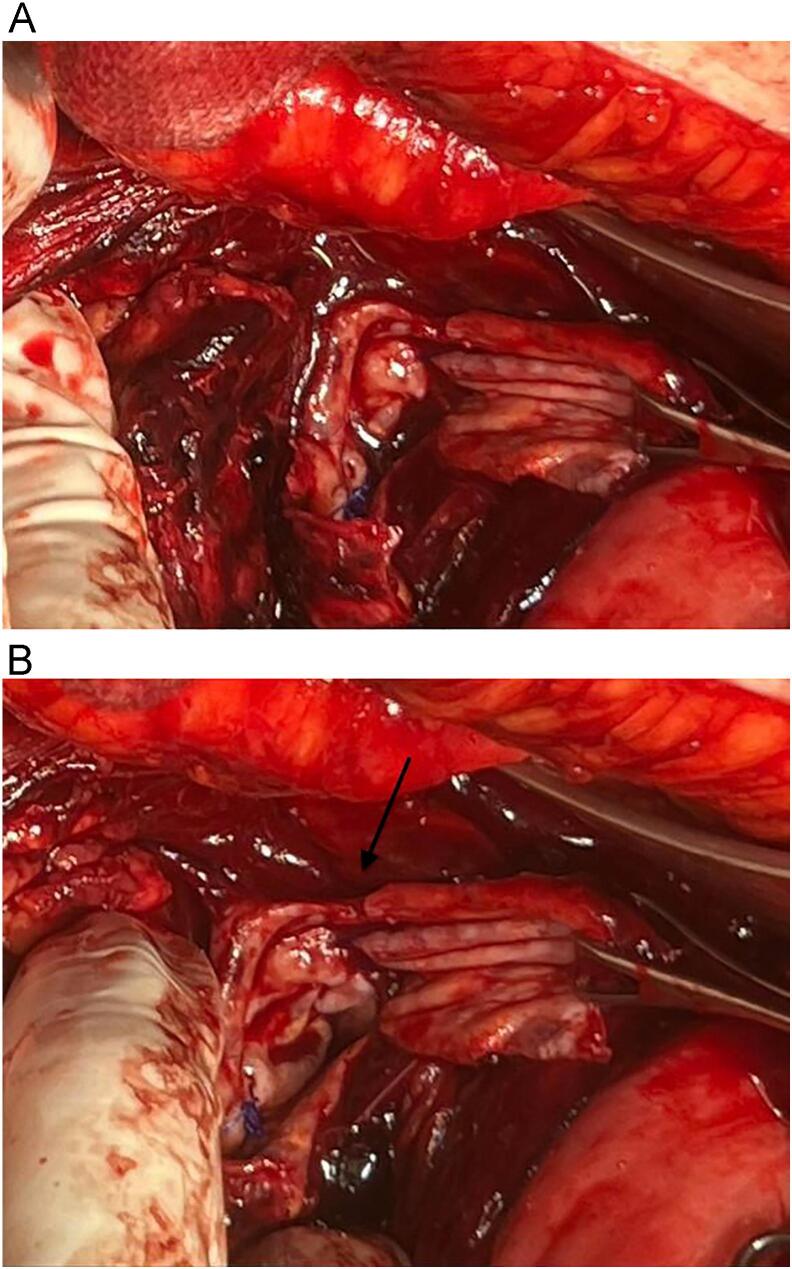
Aneurysm resection, b: arrow; aneurysm sac.

To verify occlusion of the major splenic artery and patency of the collateral arteries, selective splenic, celiac, and superior mesenteric arteries, angiograms were used as post-embolization tests. The patient underwent surgery in the hybrid operating room. Then, in laparotomy, the distal part was ligated and proximal part according to the inflated balloon, eliminating the need for extensive dissection inside the hematoma for proximal control and endangering the patient's life due to bleeding during surgery. The aneurysm was ligated and removed (Fig. 3).

The abdomen was flushed with warm, normal saline and subsequently closed upon achieving hemostasis. The patient, who remained stable, was transferred to the intensive care unit (ICU). During her ICU stay, the patient's hemodynamic parameters remained steady. Under continuous monitoring by her obstetrician and vascular surgeon, she was extubated on the second day after surgery and discharged from the hospital on the fifth postoperative day.

A simple closure of the primary splenic artery trunk does not result in major difficulties because of the spleen's strong collateral circulation. In our patient with involvement of the proximal part, we decided for an aneurysm resection without splenectomy. This approach aims to retain the spleen, a vital component of the immune system.

## 3 Discussion

A localized arterial expansion that is 50 % bigger in diameter than normal vascular size is indicative of SAA [12]. SAA is the prevailing form of arterial visceral aneurysms, constituting >60 % of total cases. Our case involved a female patient, aligning with existing literature [13].

Based on previous reports, approximately 10 % of people with SAA experienced a sudden rupture, marked by a rapid drop in blood pressure and severe abdominal pain in the upper left quadrant. The exact cause is uncertain, leading to the possibility that atherosclerosis played a key role [8,10,14].

The occurrence of a “double rupture” phenomenon, where there is an initial tamponade of splenic artery hemorrhage into the lesser sac, can be observed in up to 25 % of cases involving ruptured SAA. This phenomenon may precede the subsequent free rupture into the retroperitoneum, and in some instances, this interval can extend up to 4 days [[15], [16], [17], [18]].

Treatment is advised for all symptomatic SAAs and pseudoaneurysms. Additionally, asymptomatic patients with lesions ≥2 cm (due to the high risk of rupture), pregnant or fertile patients, those with portal hypertension, and liver transplant candidates should also receive treatment. It's important to note that there is no consensus on how to treat SAA patients, regardless of diameter [19].

The open surgical intervention is regarded as the gold standard. The most effective methods for resecting an aneurysm with an interposition bypass involve the proximal portion of the mid-splenic artery [20]. An alternative to open abdominal surgery that is less intrusive is laparoscopic with minimal excision. Nevertheless, it is not suitable for larger aneurysms and lesions with thick adhesions to adjacent tissues. Additionally, it is contraindicated for individuals who are hemodynamically unstable or at risk of rupture [21,22].

Recently, endovascular procedures such as endovascular intervention (stent or coil embolization), have also been utilized to minimize the risks of surgery and shorten the patient's hospital stay [23].

The most frequent complications of transcatheter embolization include coil migration and recanalization, which may lead to rebleeding and aneurysm rupture. Other potential complications encompass intestinal infarction, fever, splenic infarction, and abscess formation [24,25].

When the procedure ends up in embolization of the afferent artery alone due to tortuosity of the splenic artery, rebleeding can still occur due to collateral vessel flow [26].

A CT scan or an ultrasound with a Doppler should be performed to evaluate the effectiveness of the treatment as a follow-up [2].

Consequently, to prevent these problems, a thorough preprocedural examination of the aneurysm is required. Taking this into consideration, we propose to try a hybrid strategy operation with a good prognosis and lower complications. As far as we are aware, SAA's emergency hybrid management has not been used previously, thus it might be an appropriate choice for facilities equipped with hybrid operating rooms.

## 4 Conclusion

The emergency hybrid operation of SAAs in an emergent condition, which uses an endovascular balloon for inflow management and open resection, appears to be safer and more successful, as evidenced by our findings in institutions with the appropriate facilities. Following surgery, the patient was released with no procedure-related problems, such as a splenic infarction, and routine follow-ups showed a satisfactory outcome. The long-term effects of none of these therapies are known.

## Abbreviations

SAA: Splenic artery aneurysms
CBC: Complete blood count
CRP: C-reactive protein
CTA: Computed tomography angiography
ICU: Intensive care unit

## Consent

Written informed consent was obtained from the patient to publish this case report and accompanying images. On request, a copy of the written consent is available for review by the Editor-in-Chief of this journal.

## Provenance and peer review

Not commissioned, externally peer-reviewed.

## Ethical approval

Ethical approval for this study (IR.TUMS.SINAHOSPITAL.REC. 1402.115) was provided by the Ethics Committee of Tehran University of Medical Sciences, Tehran, IRAN.

## Funding

This research did not receive any specific grant from funding agencies in the public, commercial, or not-for-profit sectors.

## Guarantor

Amir Shokri.

## Research registration number

Not applicable.

## CRediT authorship contribution statement

J.S. and A.S.B. contributed to developing the idea. P.N. and F.R. contributed to the searching, extraction, and drafting of the manuscript. P.P. and A.S. and M.R.A. contributed to editing and revising the manuscript. All authors have read and agreed to the published version of the manuscript.

## Declaration of competing interest

The authors declare that they have no competing interests.
